# Dissociation of Stimulus Representation and Response Selection in Conflict Processing of Multiple Frames of Reference

**DOI:** 10.1002/pchj.70033

**Published:** 2025-06-28

**Authors:** Weizhi Nan, Zhenghan Li, Yuwei Sun, Yanlong Sun, Hongbin Wang, Qi Li, Xun Liu

**Affiliations:** ^1^ Department of Psychology and Center for Brain and Cognitive Sciences, School of Education Guangzhou University Guangzhou China; ^2^ Institute of Brain Science and Department of Physiology, School of Basic Medical Sciences Hangzhou Normal University Hangzhou China; ^3^ Beijing Forestry University Beijing China; ^4^ Institute of Artificial Intelligence, Hefei Comprehensive National Science Center Hefei Anhui China; ^5^ National Key Laboratory of Human‐Machine Hybrid Augmented Intelligence Xi'an Jiaotong University Xi'an Shanxi China; ^6^ Center for Biomedical Informatics Texas A&M University Health Science Center Houston Texas USA; ^7^ Beijing Key Laboratory of Learning and Cognition, School of Psychology Capital Normal University Beijing People's Republic of China; ^8^ CAS Key Laboratory of Behavioral Science Institute of Psychology Beijing China; ^9^ Department of Psychology University of the Chinese Academy of Sciences Beijing China

**Keywords:** conflict processing, frame of reference, response selection, stimulus representation

## Abstract

Humans use multiple frames of reference (FORs) to represent spatial information, for example, one egocentric FOR (anchored on the observer) and various intrinsic FORs (anchored on the objects in the environment). Previous studies have shown that the cognitive resource competition of FORs will lead to FOR‐based conflicts (e.g., egocentric–intrinsic, intrinsic–intrinsic) and their interactions. However, it remains unclear whether these conflicts and their interactions occur during the cognitive process stage of stimulus‐representation, response‐selection, or both. In our study, on the basis of a modified two‐cannon task, the spatial congruency and response congruency of two cannons (intrinsic FORs anchored) were manipulated to localize the two process stages of intrinsic–intrinsic conflict. The results revealed that intrinsic–intrinsic conflict was affected by both factors, indicating that response time (RT) in the spatially incongruent condition was longer than that in the spatially congruent condition and that RT in the response incongruent condition was longer than that in the response congruent condition. Furthermore, an interaction between egocentric–intrinsic and intrinsic–intrinsic conflicts was observed, showing that the egocentric–intrinsic conflict did not change between the spatially congruent and incongruent conditions but increased from the response congruent condition to the response incongruent condition. These findings suggest that intrinsic–intrinsic conflict occurs in both the stimulus‐representation and response‐selection stages, whereas egocentric–intrinsic conflict occurs only in the response‐selection stage. The two conflicts share a common conflict processing mechanism in the response‐selection stage.

## 1 Introduction

**FIGURE 1:**
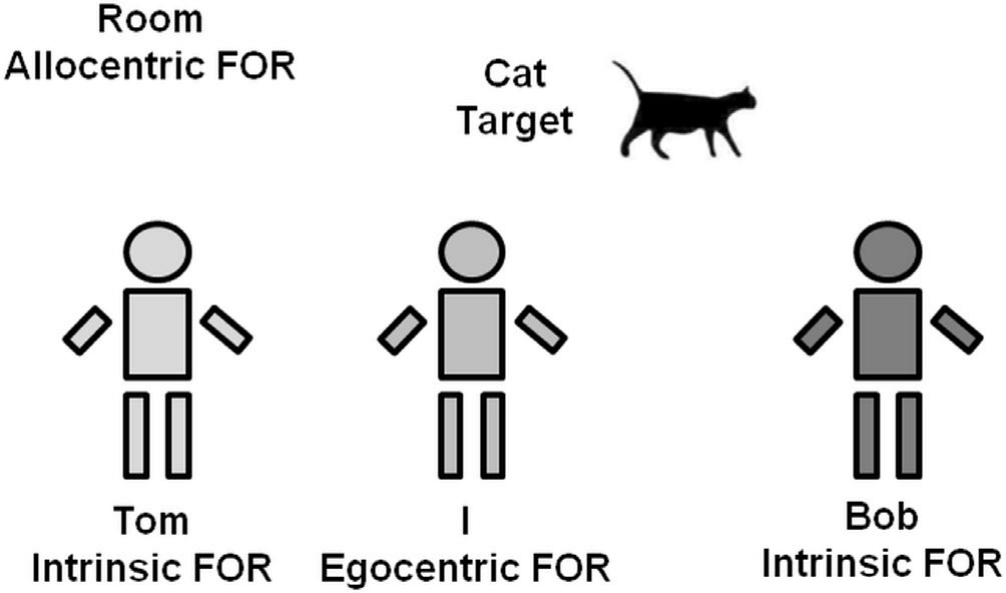
Some spatial relationships in different frames of reference. There are three individuals (Tom, I, and Bob) and a cat in a room. The cat's location can be defined in one egocentric reference system (relative to the observer self I, i.e., on the right side), two intrinsic reference systems (relative to the two other people—Tom and Bob, i.e., on Tom's right side and Bob's left side), and one allocentric reference system (relative to the room, i.e., on the room's right side).

People adopt multiple frames of reference (FORs) to represent the complex external environment (Klatzky 1998; Mou and McNamara 2002; Sun and Wang 2014; Tamborello et al. 2012; Wang et al. 2001; Zacks and Michelon 2005; Zhang et al. 2014). Adopting the psycholinguistic classification system (Levinson 1996; Sun and Wang 2014), FORs can be divided into three categories: self‐centered egocentric (anchored on the observer), environment‐centered allocentric (with the outside environment as an absolute and fixed anchor point), and environment‐centered intrinsic (with objects in the environment as relative and flexible anchors). The spatial representation changes when different FORs are chosen. For example, as shown in Figure 1, there are different FORs in the room, and the cat's reference location differs depending on the choice of FOR. The cat is on the right with respect to me (egocentric FOR), Tom (intrinsic FOR), and the room (allocentric FOR), whereas it is on the left with respect to Bob (intrinsic FOR).

According to the “frame of reference‐based map of salience” (FORMS) theory, humans represent spatial information simultaneously via multiple FORs, and human performance is determined by the interaction among all relevant FOR‐based representations (Nan et al. 2016; Sun and Wang 2010; Tamborello et al. 2012; Wang et al. 2001). Each representation is a salience map with a distinctive FOR, and these FORs jointly influence our representation and processing of spatial information. For each FOR, only highly salient objects or locations are represented and receive more attention during processing than those with low saliency on a spatial map. Therefore, while performing a task that requires a response of low‐ rather than high‐salience FOR, conflicts among these FORs may occur (Chen et al. 2012; Nan et al. 2016).

**FIGURE 2:**
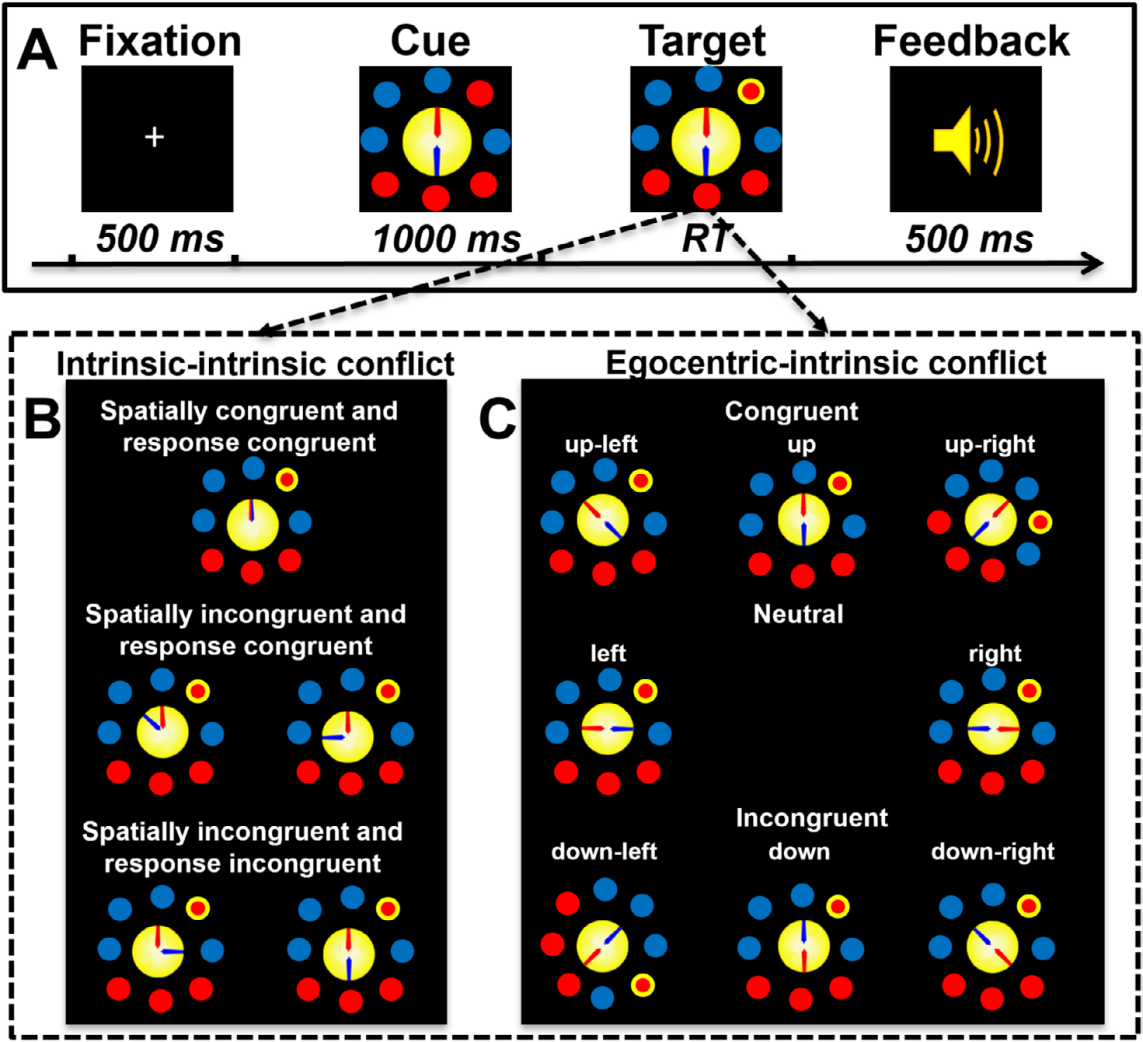
Schematic illustration of the two‐cannon task. (A) Trial procedure. At the start of the experiment, a fixation cross was displayed for 500 ms, followed by eight blue and red pellets that appeared around two colored cannons (one blue and one red) for 1000 ms; then, one of eight pellets would flash a yellow ring around it, and the participants were instructed to judge the flashing pellet location (left or right) in reference to the color‐matching cannon as quickly and accurately as possible. After 1500 ms of the target pellet display, auditory feedback indicating a correct (a shot sound) or incorrect/no (an alarm sound) response was presented for 500 ms. (B) Three two‐cannon relationship conditions were designed to test intrinsic–intrinsic conflict. (C) Three conditions of color‐matching cannon orientation were designed to test egocentric–intrinsic conflict.

In the previous cat example, if an observer is asked to judge the cat's location in Bob's egocentric FOR, he needs to take a perspective on Bob, then judge the target's location, the answer is on his left side. However, the cat's location in the observer's egocentric FOR (right side) is opposite to the location in Bob's intrinsic FOR (left side); thus, an egocentric–intrinsic conflict occurs. The cat's location is on the right side of Tom's intrinsic FOR, which is also opposite to the location in Bob's intrinsic FOR (left side); thus, an intrinsic–intrinsic conflict occurs. According to the involvement of different FORs, FOR‐based conflicts can be categorized into the following four types: egocentric‐allocentric, allocentric–intrinsic, egocentric–intrinsic, and intrinsic–intrinsic conflicts (Nan et al. 2018; Sun and Wang 2014). To investigate how humans process and resolve multiple FOR‐based conflicts, a two‐cannon task that induces multiple FOR‐based conflicts was developed (Nan et al. 2016, 2018; Tamborello et al. 2012). In this task (Figure 2A), two intrinsic FORs were anchored by two cannons, and the egocentric FOR was anchored by the subjects themselves. The observer is asked to take a perspective on a cannon with the same color as the target pellet, and then to judge the target pellet's location is on the left or right side of the color‐matching cannon. The color‐matching cannon could have the same orientation (upward) or an opposite orientation (downward) with the observer (upward). When the color‐matching cannon is facing upward, its left–right side matches that of the observer's egocentric FOR. When the color‐matching cannon is facing downward, its left–right side is opposite to that of the observer's egocentric FOR. The target pellet could be on the left or right side of the two cannons and the observer. If a target's location in the color‐matching cannon's intrinsic reference is opposite to the location in the observer's egocentric FOR, an egocentric–intrinsic conflict occurs. If a target's location in the color‐matching cannon's intrinsic FOR is opposite to the location in another color non‐matching cannon's intrinsic FOR, an intrinsic–intrinsic conflict occurs. The results revealed an intrinsic–intrinsic conflict, an egocentric–intrinsic conflict, and an interaction between them, suggesting that our brains may share a common conflict processing mechanism to address intrinsic–intrinsic and egocentric–intrinsic conflicts (Nan et al. 2016).

However, it is worth noting that there are two processing stages in the field of conflict processing: one is the early stage of stimulus representation, and the other is the late stage of response selection (Xu, Yang, Göschl, et al. 2024; Xu, Yang, Wu, et al. 2024). For example, the letter‐flanker task combines both a stimulus‐related conflict in the early stage of stimulus representation and a response‐related conflict in the late stage of response selection (Eriksen and Eriksen 1974; Treccani et al. 2009). In this task, the letters N and H are mapped to one responding key, whereas the letters K and F are mapped to the other response key; flankers could either be the same as the target (stimulus congruent and response congruent, e.g., NNNNN), different from the target but still mapped to the same response (stimulus incongruent and response congruent, e.g., KKFKK), or different from the target and mapped to the opposite response (stimulus incongruent and response incongruent, e.g., NNFNN). The stimulus incongruent and response congruent condition is thought to involve a stimulus‐related conflict between the representations of the flanker and target in the early stimulus‐representation stage relative to the stimulus congruent and response congruent condition. In addition, the stimulus incongruent and response incongruent condition induces a response‐related conflict between the responses of the flanker and target in the later response‐selection stage relative to the stimulus incongruent and response congruent condition. The results revealed that participants' response time (RT) increased across the three conditions. The subtraction method (RT [stimulus incongruent and response congruent—stimulus congruent and response congruent] and RT [stimulus incongruent and response incongruent—stimulus incongruent and response congruent]) could be used for the exploration of the stage separation of stimulus‐related and response‐related conflicts.

Following this logic, a further question to be answered is whether intrinsic–intrinsic and egocentric–intrinsic conflicts occur in the stimulus‐representation stage, in the response‐selection stage, or both. That is, whether the two conflicts are independent or share the conflict processing mechanism in two different stages.

To address the above issues, we used a revised two‐cannon task, by changing the orientations of two cannons, to manipulate two factors of spatial congruency and response congruency (Figure 2B) of two cannons to localize the process stage of spatial stimulus representation and response selection, respectively (Nan et al. 2022; Posner and Rothbart 2007; Wang et al. 2014; Xu, Yang, Göschl, et al. 2024; Xu, Yang, Wu, et al. 2024; Yan et al. 2021). The two‐cannon relationships could be categorized into three conditions: two‐cannon spatially congruent and response congruent (ScRc, 0° cannon angle; the two cannons are combined and the target location with respect to them is the same), two‐cannon spatially incongruent and response congruent (SiRc, 45° and part at a 90° cannon angle; the two cannons are spatially separate and the target occurs on the same side of the two cannons from the viewpoints of the cannons such that the target location with respect to them is the same), and two‐cannon spatially incongruent and response incongruent (SiRi, 180° and part at a 90° cannon angle; the two cannons are spatially separate, and the target occurs on the opposite sides of the two cannons from the viewpoints of the cannons such that the target locations with respect to them are opposite). Compared with the ScRc condition, a spatial congruency factor is added to the SiRc condition, which involves stimulus‐related conflict processing in the stimulus‐representation stage. Compared with the SiRc condition, a response congruency factor is added to the SiRi condition, which involves response‐related conflict processing in the response‐selection stage. According to the principle of subtraction method, the stimulus‐representation, and the response‐selection process of the intrinsic–intrinsic conflict could be extracted by the SiRc versus the ScRc condition, and the SiRi condition versus the SiRc condition, respectively (Treccani et al. 2009; Xu, Yang, Göschl, et al. 2024; Xu, Yang, Wu, et al. 2024).

Accordingly, two assumptions about the mechanism of intrinsic–intrinsic conflict and egocentric–intrinsic conflict could be made as follows: first, if intrinsic–intrinsic six conflict occurred in the stimulus‐representation stage, the response‐selection stage, or both, then it would be influenced by the factors of spatial congruency, response congruency, or the two factors, respectively. Second, if intrinsic–intrinsic conflict and egocentric–intrinsic conflict occurred in the same stage, an interaction between them in this stage would be observed.

## 2 Materials and Methods

### 2.1 Participants

The presumptive sample size in the present study was estimated by G*power 3.1 (Faul et al. 2007). For the 3 × 3 within‐factor repeated‐measures analyses of variance (ANOVAs), with a strict statistical power (1‐β) of 0.95 for detecting an effect at an alpha level of 0.05, the number of groups of 1, the number of measurements of 9, the results showed that a minimum of 22 participants was required. According to this calculation and the possible participant loss (unusual data or technical issues), the actual sample size (approximately 30) slightly exceeded the estimated sample size (22) to ensure that the actual power was reached. Thirty undergraduate and graduate students from universities in Beijing (17–29 years, average 23.1 years, 15 women) participated in the present study. All participants were right‐handed and had normal or corrected‐to‐normal vision. All the participants reported no history of neurological or psychiatric disorders or color blindness. Each participant voluntarily enrolled, signed an informed consent form prior to the experiments, and was paid 50 yuan after the experiment. The study protocol was approved by the institutional review board of the Institute of Psychology, Chinese Academy of Sciences.

### 2.2 Apparatus and Stimuli

The participants seated comfortably in a dimly lit and sound‐attenuating chamber approximately 50 cm away from a computer screen (resolution: 1024 × 768 pixels; vertical refresh rate: 75 Hz). All stimuli were composed of two cannons (one red and one blue) surrounded by eight red or blue pellets presented on the screen (Figure 2A). The stimulus could be changed according to the following five distinct dimensions: cannon angle (0°, 45°, 90°, and 180°), target color (blue and red), color‐matching cannon orientation (eight orientations: up, up‐left, left, down‐left, down, down‐right, right, and up‐right), and target location (six locations corresponding to each cannon orientation excluding the two locations that are the same as or opposite to the color‐matching cannon orientation). The stimulus presentation and manual response measurement were controlled by E‐Prime 2.0 (Psychological Software Tools Inc., Pittsburgh, PA, USA).

### 2.3 Design and Procedures

The experiment consisted of a practice phase and a formal test phase. The formal test began only after the participants completed the practice phase with 15 trials, and their accuracy was above 90%. In the formal test, 1344 trials were randomly divided into 12 blocks with 112 trials for each condition. The experimental process is illustrated in Figure 2A. At the beginning of a trial, white fixation was displayed in the center of the black screen for 500 ms; then, eight blue and red pellets appeared around two colored cannons (one blue and one red) for 1000 ms. Then, one of the eight pellets would flash a yellow ring around it to become the target. The participants were asked to judge the flashing pellet location (left or right) in reference to the color‐matching cannon as quickly and accurately as possible. If the target was on the left side of the color‐matching cannon, the participants needed to press the “Z” key of the keyboard with their left index finger; if it was on the right side, they needed to press the “?” key of the keyboard with their right index finger. After 1500 ms of the target pellet display, there was 500 ms of auditory feedback indicating a correct (a shot sound) or incorrect/no (an alarm sound) response. Each participant needed approximately 1 h to complete the entire experimental process. A 3 (intrinsic–intrinsic conflict type: ScRc, SiRc, and SiRi) × 3 (egocentric–intrinsic conflict type: congruent, neutral, and incongruent) within‐subject design was used. The intrinsic–intrinsic conflict was examined by the two‐cannon relationship (Figure 2B). According to the spatial congruency and response congruency of the two cannons, the two‐cannon relationships were categorized into three conditions: spatially congruent and response congruent (ScRc), spatially incongruent and response congruent (SiRc), and spatially incongruent and response incongruent (SiRi). The egocentric–intrinsic conflict was manipulated by the orientations of the participant and the color‐matching cannon (Figure 2C). The participant's orientation was by default pointing up, whereas the color‐matching cannon had eight orientations. In the congruent condition, when the color‐matching cannon points up (up, up‐left, or up‐right), the orientations of the color‐matching cannon and the participant are both pointed up such that the target references to them are the same. In the neutral condition, when the color‐matching cannon points horizontally (left or right), the orientations of the color‐matching cannon and the participant are orthogonal such that the target reference to them is orthogonal; in the incongruent condition, when the color‐matching cannon points down (down‐right, down, or down‐right), the orientations of the color‐matching cannon and participant are opposite such that the target reference to them is opposite.

### 2.4 Statistical Analysis

**FIGURE 3:**
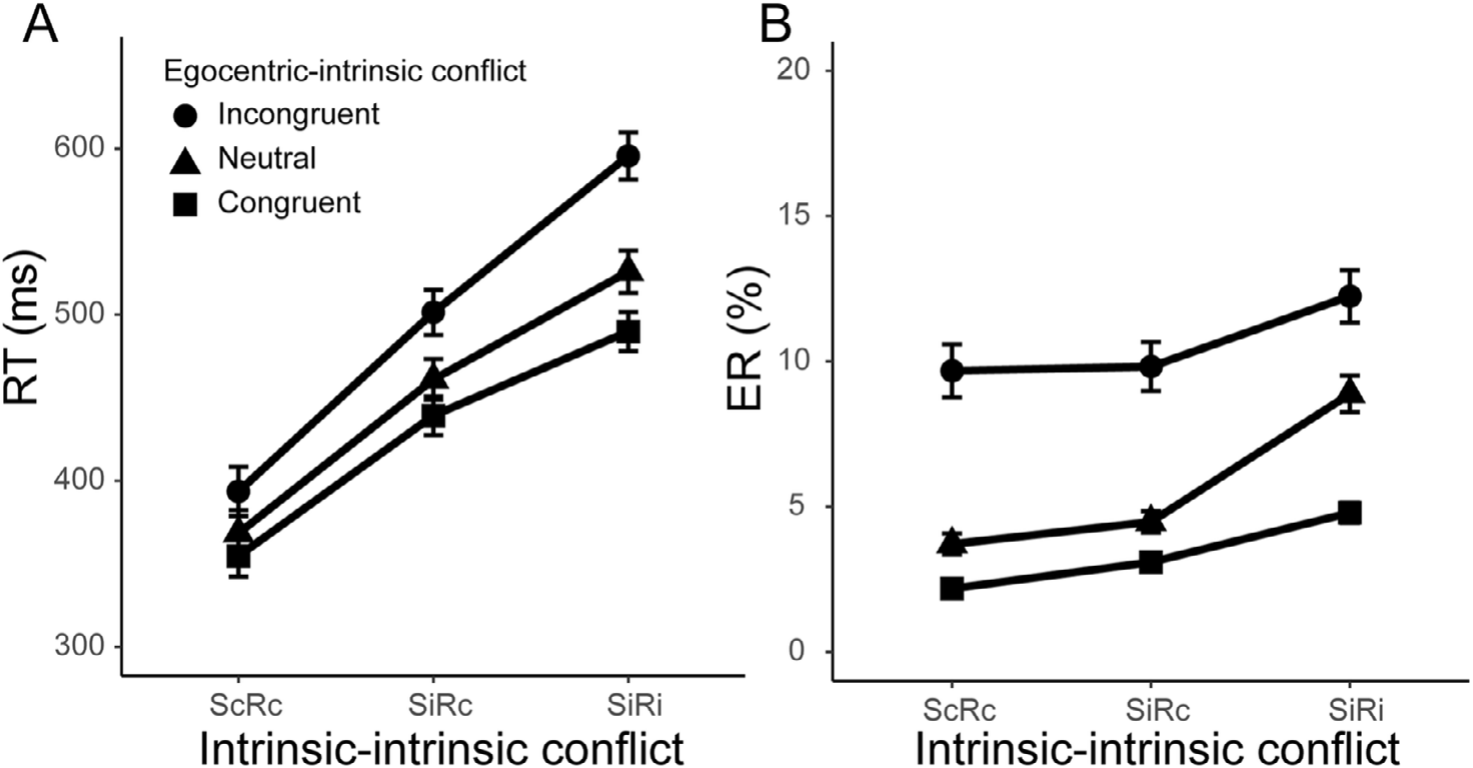
(A) RTs (with standard errors, SEs) of intrinsic–intrinsic conflict and egocentric–intrinsic conflict. (B) ERs (with SEs) of intrinsic–intrinsic conflict and egocentric–intrinsic conflict. “ScRc,” “SiRc,” and “SiRi” on the *X*‐axis are the three conditions of intrinsic–intrinsic conflict. “ScRc” means spatially congruent and response congruent; “SiRc” means spatially incongruent and response congruent; “SiRi” means spatially incongruent and response incongruent.

Trials with incorrect responses (6.6%), RTs less than 100 ms (0.4%), or RTs beyond three standard deviations in each condition (0.7%) were excluded from the RT analysis. The significance level was set at *α* < 0.05 for all ANOVAs. A 3 intrinsic–intrinsic conflict type (ScRc, SiRc, and SiRi) × 3 egocentric–intrinsic conflict type (congruent, neutral, and incongruent) repeated‐measures ANOVA was performed to analyze the RTs and ERs (Figure 3). Furthermore, for the interaction between intrinsic–intrinsic conflict and egocentric–intrinsic conflict, we split egocentric–intrinsic conflict into two sub‐effects (the facilitation effect from the subtraction between congruent and neutral conditions and the interference effect from the subtraction between incongruent and neutral conditions) to determine how they changed across the three conditions (ScRc, SiRC, SiRi) of intrinsic–intrinsic conflict (Cohen Kadosh et al. 2008). Thus, 2 one‐way ANOVAs were conducted by the factor of intrinsic–intrinsic conflict (ScRc, SiRc, SiRi) for the two sub‐effects of the egocentric–intrinsic conflict (the facilitation effect and the interference effect). Bonferroni correction was used for pairwise comparisons.

## 3 Results

With respect to the RTs (Figure 3A and Table 1), a main effect of intrinsic–intrinsic conflict was observed (*F*(2, 58) = 109.18, *p* < 0.001, *η*_p_^2^ = 0.79), and the post hoc analysis revealed that the RTs increased significantly across the three conditions (ScRc: 369 ± 25 ms, SiRc: 458 ± 23 ms, and SiRi: 523 ± 23 ms, *p*s < 0.001). A main effect of egocentric–intrinsic conflict was observed (*F*(2, 58) = 40.86, *p* < 0.001, *η*_p_^2^ = 0.59), and the post hoc analysis revealed that the RTs increased significantly across the three conditions (congruent: 418 ± 21 ms, neutral: 445 ± 24 ms, and incongruent: 486 ± 25 ms, *p*s < 0.001). There was an interaction effect between intrinsic–intrinsic and egocentric–intrinsic conflicts (*F*(4, 116) = 11.12, *p* < 0.001, *η*_p_^2^ = 0.28). Further analysis (2 one‐way ANOVAs) showed that the facilitation and interference effects of egocentric–intrinsic conflicts changed differently among the three conditions of intrinsic–intrinsic conflict. For the facilitation effect, a main effect of facilitation was observed, *F*(2, 58) = 3.70, *p* = 0.031, *η*_p_^2^ = 0.11. Post hoc analysis revealed that the facilitation effect in the SiRi (42 ± 6 ms) condition was significantly larger than that in the ScRc (15 ± 8 ms) condition (*p* = 0.041), but there was no significant difference between the ScRc and SiRc (25 ± 6 ms) conditions (*p* = 0.779) or between the SiRc and SiRi conditions (*p* = 0.351). For the interference effect, there was also a main effect of interference, *F*(2, 58) = 8.31, *p* = 0.002, *η*_p_^2^ = 0.22. Post hoc analysis revealed that the interference effect in the SiRi (65 ± 11 ms) condition was significantly larger than that in the SiRc (38 ± 6 ms) (*p* = 0.024) and ScRc (24 ± 8 ms) (*p* = 0.009) conditions, but there was no significant difference between the SiRc and ScRc conditions (*p* = 0.248).

**TABLE 1:** RTs (with SE) and ERs (with SE) of intrinsic–intrinsic conflict and egocentric–intrinsic conflict.

Egocentric–intrinsic conflict	Intrinsic–intrinsic conflict					
	RT (ms)			ER (%)		
	ScRc	SiRc	SiRi	ScRc	SiRc	SiRi
Incongruent	476 ± 21	514 ± 24	579 ± 25	10 ± 1.8	10 ± 1.7	12 ± 1.8
Neutral	429 ± 21	454 ± 24	492 ± 25	4 ± 0.7	4 ± 0.7	9 ± 1.3
Congruent	351 ± 22	366 ± 26	390 ± 27	2 ± 0.5	3 ± 0.5	5 ± 0.7

Regarding the ERs (Figure 3B and Table 1), a main effect of intrinsic–intrinsic conflict was observed (*F*(2, 58) = 15.32, *p* < 0.001, η_p_^2^ = 0.35), and the post hoc analysis revealed that the ERs in the SiRi condition (8.6% ± 1.2%) were significantly greater than those in the ScRc (5.2% ± 0.8%) and SiRc (5.8% ± 0.9%) conditions (*p*s < 0.05), while there was no significant difference between the ScRc and SiRc conditions (*p* > 0.05). A main effect of egocentric–intrinsic conflict was observed (*F*(2, 58) = 23.58, *p* < 0.001, *η*_p_^2^ = 0.45), and the post hoc analysis revealed that the ER increased significantly across the three conditions (congruent: 3.4% ± 0.4%, neutral: 5.7% ± 0.8%, and incongruent: 10.6% ± 17%, *p*s < 0.001). There was an interaction effect between intrinsic–intrinsic and egocentric–intrinsic conflicts (*F*(4, 116) = 3.14, *p* = 0.03, *η*_p_^2^ = 0.10). Further analysis (2 one‐way ANOVAs) showed that the facilitation and interference effects of egocentric–intrinsic conflicts changed differently among the three conditions of intrinsic–intrinsic conflict. For the facilitation effect, the results revealed a main effect of facilitation, *F*(2, 58) = 7.41, *p* = 0.002, *η* ^2^ = 0.20. Post hoc analysis revealed that the facilitation effect in the SiRi (4.1% ± 0.8%) condition was significantly larger than that in the SiRc (1.4% ± 0.5%) (*p* = 0.004) and ScRc (1.5% ± 0.7%) (*p* = 0.019) conditions, but there was no significant difference between the SiRc and ScRc conditions (*p* > 0.05). For the interference effect, the main effect of the interference was not significant: *F*(2, 58) = 3.01, *p* = 0.062, *η*_p_^2^ = 0.09, indicating that there was no change in the interference effect across the three conditions of intrinsic–intrinsic conflicts (ScRc: 6.0% ± 1.5%, SiRc: 5.3% ± 1.2%, SiRi: 3.4% ± 1.0%, *p*s > 0.05).

## 4 Discussion

Overall, this study used the modified two‐cannon task to explore whether intrinsic–intrinsic conflict and egocentric–intrinsic conflict have common or distinct conflict processing mechanisms in the stimulus‐representation stage, the response‐selection stage, or both. The results showed that both the spatial congruency and response congruency of the two intrinsic FORs could induce intrinsic–intrinsic conflict, which indicated that intrinsic–intrinsic conflict existed in both the stimulus‐representation and response‐selection stages. Moreover, there was an interaction effect between intrinsic–intrinsic and egocentric–intrinsic conflicts on response congruency but not on spatial congruency, suggesting that egocentric–intrinsic conflict only existed in the response‐selection stage and that the two conflicts shared a common conflict processing mechanism in the response‐selection stage. The current study provides evidence for the specificity and commonality of processing stages among different FOR‐based conflicts, further supporting the FORMS theory.

### 4.1 Intrinsic–Intrinsic Conflict Occurs in Both the Stimulus‐Representation and Response‐Selection Stages

Intrinsic–intrinsic conflict occurs in the stimulus‐representation stage. Previous studies have shown that representations of the intrinsic FOR require extra cognitive resources, whereas representations of the egocentric FOR are generated automatically (Epley et al. 2004; Sun and Wang 2014; Tamborello et al. 2012). Specifically, intrinsic FOR processing involves the recognition and memory of objects and the transformation of their intrinsic axis of orientation. These processes place considerable demands on working memory and attention, so the intrinsic FORs may be too complex to be processed automatically. Sun and Wang (2014) further noted that, compared with egocentric FOR, intrinsic FOR processing required a balance between flexibility and stability that required some attention resources. Thus, because of limited cognitive resources, competition for cognitive resources among intrinsic FORs might occur. In our study, the two intrinsic FORs are represented and processed simultaneously in the stimulus‐representation stage by manipulating the spatial congruency. Because participants do not know which intrinsic FOR would be chosen until the target appears, the brain needs to assign different attention resources to represent and process the two intrinsic FORs (Mou and McNamara 2002; Sun and Wang 2014; Tamborello et al. 2012). Therefore, compared with two cannons that were spatially congruent/overlapping, when the two cannons were spatially incongruent, that is, there was an angle between them, attention switching costs would occur between them, and the judgment time about the target location would increase.

Furthermore, intrinsic–intrinsic conflict also occurs in the response‐selection stage. Previous studies have shown the existence of a response‐related conflict caused by response congruency in the late response‐selection stage (Li et al. 2014; Spironelli et al. 2009; Treccani et al. 2009; Wang et al. 2014). When the cognitive processing caused by the presentation of stimuli conflicts with the target response position, it is necessary to suppress irrelevant information processing to respond (Lamberts et al. 1992; Lu and Proctor 1995). Smith (2011) used event‐related potential (ERP) technology and reported that response‐related conflict could increase the amplitude of the late P3 component. Wang et al. (2014) also reported that response‐related conflict (e.g., the Simon conflict caused by response congruency) occurred later than stimulus‐related conflict (e.g., Stroop conflict caused by stimulus congruency). Consistent with previous studies, our study manipulated response congruency and revealed response competition between two intrinsic FORs in the late response‐selection stage. Compared with two intrinsic FORs with the same response to the target, when two intrinsic FORs had different responses to the target, the brain required not only an enhancement to the color‐matching cannon's intrinsic FOR but also an additional inhibition to another cannon's intrinsic FOR; thus, the judgment time to the target location increased.

### 4.2 Egocentric–Intrinsic Conflict Occurs Only in the Response‐Selection Stage

Egocentric–intrinsic conflict does not occur in the stimulus‐representation stage. Compared with intrinsic FORs, the processing of an egocentric FOR is automatic and consumes minimal attention resources (Epley et al. 2004; Scheller et al. 2024; Sui et al. 2012; Sui and Humphreys 2015; Sun and Wang 2014). In our study, the egocentric FOR was task‐irrelevant and did not require subjects to deliberately carry it out. Thus, in the stimulus‐representation stage, the egocentric FOR did not compete for attention resources with the intrinsic FORs. Our results consistently showed that egocentric–intrinsic conflict was not influenced by spatial congruency.

However, egocentric–intrinsic conflict occurs in the response‐selection stage. Even if the subjects were not required to represent egocentric FOR during the task, when the target appeared, the egocentric FOR would still produce a spontaneous response to the target (Scheller et al. 2024; Sui and Humphreys 2015; Sun and Wang 2014). Following the stimulus‐representation stage, multiple responses of the FORs are combined and integrated into a final response in the response‐selection stage (Li et al. 2014; Spironelli et al. 2009; Treccani et al. 2009; Wang et al. 2014). Compared with the neutral condition, when the response of the egocentric FOR was congruent, minimal effort was needed to generate a final response, even facilitating the final response (Szűcs and Soltész 2007), showing the interference effect of egocentric–intrinsic conflict. However, when the responses between the egocentric FOR and the color‐matching cannon's intrinsic FOR were incongruent, the competition of the two FORs would enhance the response of the color‐matching cannon's intrinsic FOR and inhibit the responses of the observer's egocentric FOR, showing the interference effect of egocentric–intrinsic conflict (Cohen Kadosh et al. 2008). Furthermore, when the response produced by the color non‐matching cannon's intrinsic FOR was incongruent with that of the color‐matching cannon's intrinsic FOR, it also needed to be inhibited (Treccani et al. 2009); thus, an interaction between the intrinsic–intrinsic conflict and the egocentric–intrinsic conflict was shown in the response selection stage.

### 4.3 Theory Contribution of the Current Study

The FORMS theory (Nan et al. 2016; Sun and Wang 2014; Tamborello et al. 2012; Wang et al. 2001) posits that space is represented in the mind not once but multiple times, not unified but segmented. Each representation is a salience map with a distinctive FOR, and these FORs jointly influence our representation and processing of spatial information. Drew on Levinson's theory but unlike his theory (Levinson 1996), FORMS theory suggests spatial cognition is a basic brain function with a distinct computational goal: to understand space by identifying and emphasizing important object‐to‐self and object‐to‐object spatial relations. It emphasizes the self‐centered selection of reference systems anchored to external objects or other observers. In other words, the intrinsic FOR is not observer‐independent. Instead, its selection must first resolve self‐other conflicts and incorporate components of predictive segmentation of the allocentric representation (e.g., salience‐based processing, eliminating the need to compute the entire cognitive map). However, the FORMS theory does not elaborate in detail how different FOR‐based representations interact with each other across the cognitive process. Our study first induced stage separation to FOR‐based conflict processing, which provided further clarification and expansion of the FORMS theory concerning how people represent and process multiple FORs, and provided a more ecological angle to observe how the brain resolves the conflict. Our study revealed the specificity and commonality of processing stages among different FOR‐based conflicts. A previous study revealed that egocentric–intrinsic conflict and intrinsic–intrinsic conflict share a conflict processing mechanism (Nan et al. 2016). In the present study, we further separated the conflict process into two stages, the stimulus‐representation stage and the response‐selection stage, and further located the shared mechanism of egocentric–intrinsic conflict and intrinsic–intrinsic conflict in the response‐selection stage. Our findings, from the different FOR‐based conflicts, further suggest that resolving conflict involves a hybridized architecture of both modular and centralized cognitive control mechanisms rather than a completely domain‐specific or domain‐general processor (Liu et al. 2004; Yang et al. 2015).

### 4.4 Limitations and Future Directions

The present study's sample, comprising 30 undergraduate and graduate students from Beijing, is relatively small, which restricts the generalizability of the findings to other populations. Future research endeavors could be directed toward expanding the sample to a more diverse range of individuals to examine differences in the processing of FOR‐based conflicts (Zhang et al. 2014). The experimental design employed in this study is intricate, which may impose a cognitive load (Liu et al. 2024) and potentially influence the accurate observation of conflicts. Simplifying the experimental task, for instance, by reducing the number of cannon orientations or pellets, might enhance the replicability of the results. If subjects want to complete this task well, in addition to possessing the ability to resolve FOR‐based conflicts, they also need cognitive abilities such as perspective‐taking (Sun and Wang 2014), mental rotation (Gunderson and Hildebrand 2021), and general conflict resolution (Xu, Yang, Wu, et al. 2024). Notably, conflict resolution hinges on the cognitive control mechanism, which is anchored in a neural network involving the anterior cingulate cortex (ACC), dorsolateral prefrontal cortex (dlPFC), and sensory cortex (Kim et al. 2010; Li et al. 2017). This mechanism selectively enhances neural representations related to tasks while suppressing the neural activation of interfering information, ultimately achieving effective conflict resolution (Li et al. 2020; Polk et al. 2008). This cognitive control mechanism provides a ground for the higher‐level adaptive cognitive strategies. Existing evidence shows that cognitive conflict resolution training can significantly enhance an individual's cognitive control efficiency, prompting participants to develop more flexible cognitive strategies (Blair and Razza 2007; Karbach and Verhaeghen 2014; Rueda et al. 2005). By predicting interference sources and dynamically allocating cognitive resources, they can optimize multitasking abilities. Blair and Razza (2007) found that the inhibitory function of cognitive control was a prominent correlate of both early math and reading ability. Future research could further develop this simplified task and apply it to other domains and age groups, such as assessing and training spatial cognitive abilities in young or elderly populations.

## 5 Conclusion

The present study investigated conflict processing among multiple FORs. Our experimental task separated the two factors of spatial congruency and response selection to divide intrinsic—intrinsic FOR‐based conflict processing into the two subprocesses of stimulus representation and response selection. Our results support dissociation processing in intrinsic–intrinsic and egocentric–intrinsic conflicts. In the stimulus‐representation stage, the egocentric FOR is automatically processed, while different intrinsic FORs compete for attention resources according to the input information. Then, in the response‐selection stage, different responses are generated from each FOR, and a shared conflict processing mechanism works for the intrinsic–intrinsic and egocentric–intrinsic conflicts to generate a final response.

## Conflicts of Interest

The authors declare no conflicts of interest.

## Data Availability

The anonymized data, stimuli and preprocessing/analysis details are available upon request.
